# *Aspergillus*-bees: A dynamic symbiotic association

**DOI:** 10.3389/fmicb.2022.968963

**Published:** 2022-09-07

**Authors:** Andrea Becchimanzi, Rosario Nicoletti

**Affiliations:** ^1^Department of Agricultural Sciences, University of Naples Federico II, Portici, Italy; ^2^Council for Agricultural Research and Economics, Research Centre for Olive, Fruit and Citrus Crops, Caserta, Italy

**Keywords:** Aspergillaceae, saprophytic fungi, fungal entomopathogens, pollinators mycobiota, mycotoxins, bee immunity

## Abstract

Besides representing one of the most relevant threats of fungal origin to human and animal health, the genus *Aspergillus* includes opportunistic pathogens which may infect bees (Hymenoptera, Apoidea) in all developmental stages. At least 30 different species of *Aspergillus* have been isolated from managed and wild bees. Some efficient behavioral responses (e.g., diseased brood removal) exerted by bees negatively affect the chance to diagnose the pathology, and may contribute to the underestimation of aspergillosis importance in beekeeping. On the other hand, bee immune responses may be affected by biotic and abiotic stresses and suffer from the loose co-evolutionary relationships with *Aspergillus* pathogenic strains. However, if not pathogenic, these hive mycobiota components can prove to be beneficial to bees, by affecting the interaction with other pathogens and parasites and by detoxifying xenobiotics. The pathogenic aptitude of *Aspergillus* spp. likely derives from the combined action of toxins and hydrolytic enzymes, whose effects on bees have been largely overlooked until recently. Variation in the production of these virulence factors has been observed among strains, even belonging to the same species. Toxigenic and non-toxigenic strains/species may co-exist in a homeostatic equilibrium which is susceptible to be perturbed by several external factors, leading to mutualistic/antagonistic switch in the relationships between *Aspergillus* and bees.

## Introduction

Pollination by insects is one of the most important mechanisms involved in maintenance and promotion of biodiversity on Earth and has a direct effect on agricultural activities, contributing to about one third of the global crop production (Klein et al., 2007). Wild bee species, central to the agro-ecosystem service of pollination (Garibaldi et al., 2013), have been declining in many parts of the world (Goulson et al., 2015), attracting the attention of the public opinion, which stimulated government policies aimed at protecting these species (Laursen, 2015).

Due to the great variety of the visited floral species, honey bees (*Apis mellifera* L.) are at the center of several pollination networks and represent the most widespread pollinating species. Although honey bee world population has increased in recent decades, along with beekeeping activities (Geldmann and González-Varo, 2018), a high proportion of colony losses has been reported at a local scale by several monitoring programs (Kulhanek et al., 2017). Besides climatic and anthropogenic factors, colony losses have been related to the incidence of biotic adversities caused by parasites and pathogens, including protozoans, viruses, bacteria and fungi (Genersch et al., 2010; Schwarz et al., 2015; Flores et al., 2021; Ribani et al., 2021; Lannutti et al., 2022; Schäfer et al., 2022). Among the latter pathogens, the most relevant are represented by microsporidia (*Nosema* spp.; Grupe and Quandt, 2020) and *Ascosphaera apis* (Eurotiomycetes, Ascosphaeraceae), the causal agent of chalkbrood (Aronstein and Murray, 2010). Interactions between bees and other fungi, particularly species in the genus *Aspergillus* (Eurotiomycetes, Aspergillaceae), are less definite, and range from mutualistic to parasitic.

*Aspergillus* spp. are ubiquitous in terrestrial habitats due to their ability to disperse globally with air currents and to grow on many different substrates. These fungi are commonly isolated from soil, particularly from plant litter. Indeed, many species of *Aspergillus* can abundantly grow as saprophytes on decaying vegetation and are adapted for the degradation of complex plant polymers (Bennett, 2010). Thus, the association of several *Aspergillus* species with bees and bee products, particularly with pollen, is not surprising. Pollen represents an entry for fungal pathogens in the hive. Indeed, spores of *Aspergillus* spp. may contaminate pollen on plants (Sainger et al., 1978; Gilliam et al., 1989); once collected, stored and consumed by bees, these spores reach the gut, which is the primary site of infection for bee pathogens (Foley et al., 2014). According to the prevalent point of view, several *Aspergillus* spp. are considered as opportunistic pathogens which may infect bees in all developmental stages (Foley et al., 2012). Intriguingly, infection with *Aspergillus* species can provoke symptoms very similar to the colony collapse disorder firstly described in 2006, with no or very few adult bees remaining in the hive (Burnside, 1930; Hamzelou, 2007; Leska et al., 2021). Nevertheless, *Aspergillus flavus*, the causal agent of stonebrood, is considered of minor importance and is poorly studied in the framework of the honey bee pathosphere (Foley et al., 2014; Schwarz et al., 2015).

On the other hand, *Aspergillus* spp. are considered one of the most relevant threats of fungal origin to human and animal health (Seyedmousavi et al., 2015). Indeed, some *Aspergillu*s species are mycotoxigenic and represent a sanitary risk related to contamination in the feed and food production chains (Ráduly et al., 2019). Moreover, they are zoonotic pathogens that can cause aspergillosis in humans, with symptoms ranging from allergic reactions to true infections of the respiratory system, primarily in immune-compromised patients or those already suffering from other lung diseases (Dagenais and Keller, 2009). Considering the known effects on human health of species such as *A. fumigatus* and *A. flavus* (de Graaf et al., 2008), an accurate knowledge of the association between honey bees and *Aspergillus* spp. is also relevant for the safety of beekeepers.

The present work is aimed at reviewing the currently available literature concerning the interactions between *Aspergillus* species and both wild and managed bees, focusing on pathogenic and mutualistic interactions.

## Species of *Aspergillus* reported in association with bees and bee products

The genus *Aspergillus* consists of six subgenera and 18 sections, which accommodate over 250 species (Gams et al., 1986; Tsang et al., 2018). Microscopic examination of conidial structures and macroscopic characteristics of the colony (texture, growth rate, degree of sporulation, conidial and mycelial colors) can be used for species differentiation. However, DNA sequencing and phylogenetic analysis of *calmodulin* and *β-tubulin* loci have become the gold standard for accurate identification at the species level (Tsang et al., 2018; Houbraken et al., 2020). Recent studies reported that subtle phenotypic variation between cryptic *Aspergillus* spp. can have strong implications with their pathogenicity toward bees, highlighting the importance of an accurate identification of the isolates (Foley et al., 2014).

Based on data available in literature and GenBank, so far at least 30 different species of *Aspergillus* have been isolated from bees (Table 1), mostly belonging to the sections *Flavi, Fumigati* and *Nigri*. In particular, honey bees resulted associated with 25 identified *Aspergillus* species, while wild bees resulted associated with 14 *Aspergillus* spp. However, one should consider that many studies cited in Table 1 were conducted before the more recent description of new species, and before the spread of DNA sequencing and other accurate identification methods, such as those based on specific antibodies (Schubert et al., 2018). Thus, species such as *A. nomius*, which was described in 1987 and is phenotypically similar to *A. flavus* (Kurtzman et al., 1987), are probably underrepresented. In the cited studies, most of which were conducted in North and South America, the sources of isolation were highly diverse, with a prevalence of mummified and diseased brood, dead and living adults (Table 1). Notably, in the cited studies external sterilization of the samples has been rarely carried out, making it impossible to establish if the isolated fungi were developing internally, or were just contaminating the integument.

**Table 1:** Occurrence of *Aspergillus* species reported as bee associates.

*Aspergillus* species	Bee species	Source	Location	Reference
*A. alliaceus*	*Nomia melanderi*	Brood cells	Michigan, United States	Burnside (1930)
*A. amstelodami* (= *A. montevidensis*?)	*Apis mellifera*	Gut of adult workers	Arizona, United States	Gilliam and Prest (1972)
*A. aureoterreus*	*N, melanderi*	Brood	Northwestern United States	Batra et al. (1973)
	*A. mellifera*	Hive	Michigan, United States	Burnside (1930)
	*Nomia triangulifera*	Brood	Northwestern United States	Batra et al. (1973)
*A. caelatus*	Stingless bee	Unknown	Malaysia	GenBank: MW040902
*A. calyptratus*	*A. mellifera*	Dead adults	Michigan, United States	Burnside (1930)
*A. candidus*	*A. mellifera*	Dead adults	Michigan, United States	Burnside (1930)
	*A. mellifera*	Adult gut	Poland	Kaznowski et al. (2005)
*A. clavatus*	*A. mellifera*	Dead adults	Michigan, United States	Burnside (1930)
*A. flavus*	*Augochlora pura*	Dead adults	Michigan, United States	Burnside (1930)
	*A. mellifera*	Diseased brood, comb	Michigan, United States	Burnside (1930)
	*A. mellifera*	Mummified larvae	Northwestern United States	Batra et al. (1973)
	*N. melanderi*	Diseased prepupae	Northwestern United States	Batra et al. (1973)
	*Anthophora pacifica*	Diseased brood	Northwestern United States	Batra et al. (1973)
	*Anthophora occidentalis*	Diseased brood	Northwestern United States	Batra et al. (1973)
	*N. triangulifera*	Diseased brood	Northwestern United States	Batra et al. (1973)
	*Lasioglossum zeiphyrum*	Diseased brood	Northwestern United States	Batra et al. (1973)
	*Megachile rotundata*	Diseased adults; crop; excreta	Northwestern United States	Batra et al. (1973)
	*A. mellifera*	Gut of adult workers	Arizona, United States	Gilliam et al. (1974)
	*Apis florea*	Mummified brood	Iran	Alizadeh and Mossadegh (1994)
	*A. mellifera*	Mummified brood; healthy larvae and adults	Egypt	Shoreit and Bagy (1995)
	*A. mellifera*	Gut of adult workers	Slovakia	Kačániová et al. (2012)
	*A. mellifera*	Homogenized larvae; adult gut	England	Foley et al. (2014)
	*A. mellifera adansonii*	Adult gut and integument	Nigeria	Ayo Fasasi (2018)
	*A. mellifera*	Adult hemolymph	Italy	DellaGreca et al. (2019)
	*A. mellifera*	Homogenized larvae and adults	Turkey	Bayrakal et al. (2020)
A. fresenii^*^	*N. melanderi*	Diseased larvae and prepupae	Nortwestern United States	Batra et al. (1973)
	*A. mellifera*	Hive	Michigan, United States	Burnside (1930)
*A. fumigatus*	*N. melanderi*	Diseased prepupae	Northwestern United States	Batra et al. (1973)
	*A. mellifera*	Diseased adults and brood	Michigan, United States	Burnside (1930)
	*M. rotundata*	Excreta of chalkbrood-infected larvae	Alberta, Canada	Inglis et al. (1993)
	*A. florea*	Mummified brood	Iran	Alizadeh and Mossadegh (1994)
	*A. mellifera*	Mummified brood; healty larvae and adults	Egypt	Shoreit and Bagy (1995)
	*A. mellifera*	Homogenized larvae; adult gut; hive airborne	England	Evison et al. (2013), Foley et al. (2014)
	*A. mellifera adansonii*	Adult gut and integument	Nigeria	Ayo Fasasi (2018)
	*A. mellifera*	Homogenized larvae and adults	Turkey	Bayrakal et al. (2020)
*A. glaucus*	*A. mellifera*	Mummified adults and larvae	Michigan, United States	Burnside (1930)
	*M. rotundata*	Larval cadavers	Saskatchewan, Canada	Goerzen (1991)
*A. nidulans*	*A. mellifera*	Diseased adult and brood	Michigan, United States	Burnside (1930)
*A. niger*	*A. mellifera*	Mummified adults and larvae	Michigan, United States	Burnside (1930)
	*A. mellifera*	Queen larva; Gut of adult workers	Arizona, United States	Gilliam and Prest (1972), Gilliam et al. (1974)
	*Anthophora abrupta*	Brood	Maryland, United States	Norden and Scarbrough (1982)
	*M. rotundata*	Living adults; larval cadavers; spoiled cell; larval excreta	Saskatchewan, Canada	Goerzen (1991)
	*A. florea*	Mummified brood	Iran	Alizadeh and Mossadegh (1994)
	*A. mellifera*	Mummified brood; healty larvae and adults	Egypt	Shoreit and Bagy (1995)
	*Melipona subnitida*	Dead adults	Brazil	Morais et al. (2013)
	*A. mellifera*	Homogenized larvae; adult gut	England	Foley et al. (2014)
	*A. mellifera adansonii*	Integument; gut	Nigeria	Ayo Fasasi (2018)
	*A. mellifera*	Gut	Italy	Callegari et al. (2021)
	*A. florea*	Gut	Saudi Arabia	Callegari et al. (2021)
	*A. mellifera jemenitica*	Gut	Saudi Arabia	Callegari et al. (2021)
*A. nomius*	*A. mellifera*	Adult gut	England	Foley et al. (2014)
	*Bombus transversalis*	Floral visiting adults (abdomen)	Amazonas, Brazil	Massi et al. (2015)
	*Centris denudans*	Floral visiting adults (abdomen)	Amazonas, Brazil	Massi et al. (2015)
	*Centris ferruginea*	Floral visiting adults (abdomen)	Amazonas, Brazil	Massi et al. (2015)
	*Epicharis flava*	Floral visiting adults (abdomen)	Amazonas, Brazil	Massi et al. (2015)
	*Xylocopa frontalis*	Floral visiting adults (abdomen)	Amazonas, Brazil	Massi et al. (2015)
*A. ochraceopetaliformis*	Bees	Unknown	Egypt	GenBank: MN966663
*A. ochraceus*	*A. mellifera*	Diseased adults and brood	Michigan, United States	Burnside (1930)
	*A. mellifera*	Mummified brood; healty larvae and adults	Egypt	Shoreit and Bagy (1995)
	*A. mellifera*	Hive airborne	England	Foley et al. (2014)
	*A. mellifera*	Adult midgut	Maryland, United States	GenBank: MT472089
*A. oryzae*	*A. mellifera*	Mummified brood	Egypt	Shoreit and Bagy (1995)
	*A. mellifera*	Homogenized larvae; hive airborne	England	Foley et al. (2014)
*A. parasiticus*	*N. melanderi*	Diseased pupae and prepupae	Northwestern United States	Batra et al. (1973)
	*A. mellifera adansonii*	Adult gut and integument	Nigeria	Ayo Fasasi (2018)
*A. phoenicis*	*A. mellifera*	Adult gut	England	Foley et al. (2014)
*A. proliferans*	*A. mellifera*	Larva	South Africa	GenBank: MK451496
*A. rugulosus*	*A. mellifera*	Larval faeces	Arizona, United States	Gilliam and Prest (1987)
*A. sclerotiorum*	*A. mellifera*	Adult gut	England	Foley et al. (2014)
*Aspergillus* sp.	*A. mellifera*	Adult gut	Poland	Kaznowski et al. (2005)
	*Bombus griseocollis*	Adults (abdomen)	Ontario, Canada	Macfarlane (1976)
	*M. subnitida*	Dead adults	Brazil	Morais et al. (2013)
	*Nomia oxybeloides*	Cell wall and faeces	India	Batra (1966)
	*Osmia cornifrons*	Adults (abdomen)	New York, United States	Hedtke et al. (2015)
	*Osmia lignaria*	Whole foragers	California, United States	Cohen et al. (2020)
	*A. mellifera*	Homogenized foragers	China	Ye et al. (2021)
	*A. mellifera*	Faeces	Australia	GenBank: MK402099
*A. subversicolor*	Bee	Unknown	South Korea	GenBank: MZ687463
*A. sydowii*	*A. mellifera*	Dead adults; combs	Michigan, United States	Burnside (1930)
	*A. mellifera*	Gut of adult workers	Arizona, United States	Gilliam et al. (1974)
	*M. rotundata*	Larval excreta	Alberta, Canada	Inglis et al. (1993)
*A. tamarii*	*N. melanderi*	Faeces; all stages	Northwestern United States	Batra and Bohart (1969)
*A. terreus*	*A. mellifera*	Gut	Arizona, United States	Gilliam et al. (1974)
	*M. rotundata*	Larval excreta	Alberta, Canada	Inglis et al. (1993)
	*M. subnitida*	Dead adults	Brazil	Morais et al. (2013)
	*A. mellifera*	Gut	Italy	Callegari et al. (2021)
	*A. cerana indica*	Unknown	India	GenBank: KY800395
*A. tubingensis*	*A. mellifera*	Adult gut	England	Foley et al. (2014)
	*A. mellifera*	Chalkbrood mummies	China	Cheng et al. (2022)
*A. unguis*	*A. florea*	Gut	Saudi Arabia	Callegari et al. (2021)
*A. ustus*	*N. melanderi*	Cell content	Northwestern United States	Stephen (1959)
*A. versicolor*	*A. mellifera*	Bees; hive	Michigan, United States	Burnside (1930)
	*M. rotundata*	Pre-defecation larvae	Alberta, Canada	Inglis et al. (1993)
	*A. mellifera*	Homogenized larvae	England	Foley et al. (2014)

* This species originally reported as *A. sulphureus*.

Other arthropods associated with bees are considered as vehicles of *Aspergillus* species. *Aspergillus niger* and *A. flavus* have been found on the surface of females of *Varroa destructor* (Parasitiformes, Varroidae), indicating that this parasitic mite can be a vector for their spread in hives (Benoit et al., 2004). Whether or not *V. destructor* itself can be damaged by these fungi requires further assessments, considering that reproduction of another parasitic mite, *Imparipes apicola* (Acariformes, Scutacaridae), has been reported to be inhibited by *A. flavus* (Cross and Bohart, 1992). Another potential vector of *A. flavus* is the wax moth *Galleria mellonella* (Lepidoptera, Pyralidae), which is a common beekeeping pest. Indeed, a polyethylene-degrading strain of *A. flavus* has been recently isolated from the gut of *G. mellonella*, revealing a certain degree of plasticity of this fungal species in terms of adaptive capacity to different pH conditions, from the acid gut of bees to the extremely alkaline gut of Lepidoptera (Zhang et al., 2020). Moreover, *A. flavus* and *A. fumigatus* have been isolated from dead adults and living larvae of *Vespula* spp., which are well known predators of bees (Glare et al., 1996; Quinn et al., 2018; Loope et al., 2019), suggesting a likely mechanism of fungal spores transmission from bees to wasps.

The close association of *Aspergillus* spp. with honey bees is confirmed in the analysis of bee provisions, such as pollen (Gilliam et al., 1989). Concerning species, *A. flavus, A. fumigatus, A. niger, A. terreus* and *A. versicolor* were isolated from corn pollen, *A. niger* again from almond pollen (Gilliam et al., 1989), while *A. flavus, A. luchuensis* (= *A. niger*), *A. nidulans, A. sulphureus* (= *A. fresenii*) and *A. versicolor* were isolated in pollen collected from three herbaceous annual plants (Sainger et al., 1978). By manipulating and storing pollen inside the hive, bees alter its mycoflora composition, which is likely the result of microbial inoculations by bees and chemical changes resulting from additions of honey sac contents and secretions of glands, as well as microbial fermentation, which allow some fungal species, but not others, to survive (Gilliam et al., 1989). As a whole, *Aspergillus* spp. have a higher incidence in analysis concerning bee bread when compared to corbicular pollen in both *A. mellifera* and *A. cerana* (Table 2), which may indicate that contamination of bee bread is internal to the hive rather than deriving from the pollen sources (Gilliam et al., 1989; Disayathanoowat et al., 2020). When commercialized, pollen may still contain *A. flavus* spores as reported by several studies (González et al., 2005; Bucio Villalobos et al., 2010; Deveza et al., 2015), highlighting the potential risk for human health in bee pollen consumption due to the high contamination level by these moulds and their mycotoxins. As a matter of fact, it has been demonstrated that bee pollen is a substrate stimulating production of ochratoxin A by *A. ochraceus* (Medina et al., 2004); this mycotoxin is highly cytotoxic and is reported for insecticidal effects (Boguś et al., 2021). A few studies showed contamination of honey. In Northern Italy, *A. flavus* and *A. japonicus* have been identified in a shotgun sequencing of DNA contained in honey (Bovo et al., 2020). In Turkey, *A. flavus* and *A. fumigatus* were, respectively, found in 4.4 and 6.4% of the honey samples examined (Dümen et al., 2013). The species *A. flavus, A. niger, A. fumigatus, A. candidus, A. terreus, A. versicolor, A. ochraceus* were recovered in an investigation carried out on 50 honey samples in Slovakia (Kačániová et al., 2009), while the first two were found in honey samples analysed in Brazil (Pires et al., 2015). In the latter country, *A. flavus* was also reported to occur in honey of the stingless bee *Melipona scutellaris* (Gois et al., 2010).

**Table 2:** *Aspergillus* species reported from pollen collected by honey bees.

*Aspergillus* species	Location	Reference
*A. amstelodami* (= *A. montevidensis*?)	Arizona, United States	Gilliam et al. (1989)
*A. carbonarius*	Argentina	Patiño et al. (2005)
	Brazil	Deveza et al. (2015)
	Spain	Patiño et al. (2005)
*A. flavus*	Arizona, United States	Gilliam et al. (1989)
	Egypt	Shoreit and Bagy (1995)
	Argentina	Patiño et al. (2005)
	Slovakia	Kačániová et al. (2009)
	Mexico	Bucio Villalobos et al. (2010)
	Brazil	Deveza et al. (2015)
	Taiwan	Hsu et al. (2021)
*A. fumigatus*	Egypt	Shoreit and Bagy (1995)
	Spain	Patiño et al. (2005)
	Slovakia	Kačániová et al. (2009)
	Brazil	Deveza et al. (2015)
*A. japonicus*	Egypt	Shoreit and Bagy (1995)
*A. niger*	Arizona, United States	Gilliam et al. (1989)
	Egypt	Shoreit and Bagy (1995)
	Argentina	Patiño et al. (2005)
	Slovakia	Kačániová et al. (2009)
	Brazil	Deveza et al. (2015)
	Spain	Patiño et al. (2005)
	Georgia	Janashia et al. (2018)
	France	GenBank: KY886458
*A. ochraceus*	Argentina	Patiño et al. (2005)
	Slovakia	Kačániová et al. (2009)
	Brazil	Deveza et al. (2015)
	Spain	Patiño et al. (2005)
*A. oryzae*	Brazil	Deveza et al. (2015)
*A. parasiticus*	Argentina	Patiño et al. (2005)
	Spain	Patiño et al. (2005)
*A. terreus*	Slovakia	Kačániová et al. (2009)
	Brazil	Deveza et al. (2015)
*A. tubingensis*	Argentina	Patiño et al. (2005)
	Spain	Patiño et al. (2005)
*A. ustus*	Arizona, United States	Gilliam et al. (1989)
*A. versicolor*	Slovakia	Kačániová et al. (2009)
	Brazil	Deveza et al. (2015)
*Aspergillus* sp.	Slovakia	Kačániová et al. (2009)
	Mexico	Bucio Villalobos et al. (2010)
	Thailand	Sinpoo et al. (2017)
	Germany	Friedle et al. (2021)

## Pathogenic interactions

### Pathogenic interactions in honey bees

In 1896 a new disease was described in Texas which was called ‘pickled brood’ or ‘white fungus’, caused by an approximately described *Aspergillus pollini*, which was found to occur on both larvae and adults of *A. mellifera* (Burnside, 1930). In Europe, the incidence of *A. flavus* as the causal agent of stonebrood was already known at the beginning of the 20^th^ century (Burnside, 1930). Recent findings indicate that different species of *Aspergillus* can be pathogenic to bees (Foley et al., 2014). A comprehensive study carried out in England showed that, out of 10 species recovered in apiaries (Table 1), three species (*A. flavus*, *A nomius* and *A. phoenicis*) resulted pathogenic to honey bee larvae; as tested for pathogenicity towards adult bees, following diet administration, *A. flavus* proved to be pathogenic at all the tested doses, while *A. niger* and *A. fumigatus* were not infectious (Foley et al., 2014).

*Aspergillus* spp. are known to cause stonebrood, turning honey bee broods into hard mummies. As discussed above, one of the main sources of *Aspergillus* in the hive is probably pollen, as nectar is not thought to significantly harbor fungal conidia (González et al., 2005). Conidia present in the air may also colonize hive substrates saprophytically and be transmitted via physical contact or food sharing by adult bees (Foley et al., 2014). In these ways conidia may in turn be fed to larvae in the cells, where infection through the alimentary tract occurs. Spores germinate in the gut leading to an invasive mycosis and host death (Burnside, 1930). Although this is likely the primary entry point, other infection routes may occur. Indeed, external infection of larvae by cuticle penetration is possible but rare (Burnside, 1930). The ectoparasitic mite *V. destructor* may also potentially act as vector of *Aspergillus* spp., facilitating the infection through the opening of feeding wounds on the bee integument (Benoit et al., 2004). When infection occurs through wounds on the cuticle rather than through ingestion, physical barriers are by-passed and usually death is more rapid (Burnside, 1930; Leger et al., 2000). Symptoms of aspergillosis in the brood were accurately described in one of the first published studies about fungal diseases of bees (Burnside, 1930). After few hours from infection, the larvae show increased firmness and dryness. Then, a collar of hyphae emerges from the sutures around the head, and a white mycelium covers the integument. Before mummy formation, a colored sporulation usually occurs, starting from the posterior part of the abdominal tergites (Burnside, 1930).

*Aspergillus* spp. can also infect adult bees, although in this case germination of spores within the alimentary tract is the only way of infection to be considered. Indeed, the experimental application of spores and germinated conidia on the body surface of adult bees did not lead to mycosis (Burnside, 1930). The first sign of infection by *Aspergillus* in adults is restlessness, followed by weakness and paralysis. In artificially infected colonies, bees start crawling and try to leave the hive by flying, usually dying at a considerable distance (Burnside, 1930). This can be interpreted as a self-isolation behavior aimed at limiting disease transmission within the colony (Stockmaier et al., 2021).

Aspergillosis is not limited to *Apis mellifera*, but can also affect other *Apis* species. Indeed, *Aspergillus* spp. were frequently found in association with drone broods of *Apis florea* and may represent a potential threat to this bee. Out of 600 mummies sectioned and examined microscopically, 252 (42%) were infected with *A. flavus*, 138 (23%) with *A. niger*, 72 (12%) with *A. fumigatus* and 102 (17%) with either two or three of these species (Alizadeh and Mossadegh, 1994).

### Pathogenic interactions in wild bees

*Aspergillus* spp. infections may impact wild bees too. However, only a handful of mycological studies focusing on wild bees have been carried out so far. In their seminal work Batra et al. (1973) investigated fungal occurrence in the alkali bee (*Nomia melanderi*). *Aspergillus flavus, A. tamarii,* and *A. sulphureus* were isolated from all samples in all sampling periods; the first two species, in that order, were also the most abundant species in 16 of the 20 sites investigated, and they caused the heaviest damage to bees (Batra et al., 1973). *Aspergillus alliaceus* was another species occurring at some extent. *Aspergillus flavus, A. tamarii, A. aureoterreus* attacked larvae and prepupae and killed 15.68% of alkali bee population, with *A. flavus* being the most common. However, the presence of *Aspergillus* spp. in alkali bee nest cells does not necessarily result in an invasion of the larvae. Healthy prepupae are frequently found to be completely surrounded by mycelium growing from the faecal material (Batra et al., 1973). Interestingly, when living prepupae and pre-defecating mature larvae of alkali bees were plated with *A. flavus* for 24 h, spore germination was inhibited over a 1.5–3 cm zone surrounding each larva (Batra et al., 1973). The interesting practice of sealing cells containing infected broods with compact soil has also been reported in this species, emphasizing the protective role of cleaning practices in bee colonies (Batra and Bohart, 1969).

Also Megachilidae seem to be affected by *Aspergillus*, as attested by the occurrence of *A. glaucus* and *A. niger* in larval cadavers of *Megachile rotundata* (Goerzen, 1991). Moreover, a recent work reported the occurrence of *Aspergillus* sp. in adult workers of *Osmia lignaria* (Cohen et al., 2020; Table 1).

### Mycotoxins and other virulence factors

Besides causing a direct damage and depleting important nutrients, fungal entomopathogens may be lethal to insects also by producing toxic secondary metabolites. In *Aspergillus*, the production of these compounds is largely influenced by the substrate and growth conditions (Vega and Kaya, 2012; Salvatore et al., 2018; Frisvad et al., 2019). Several *Aspergillus* metabolites revealed antiinsectan effects, resulting in competitive biocenotic interactions. Uka and colleagues (2020) delineated different groups of secondary metabolites produced by *A. flavus* with a known antiinsectan effect: polyketides (aflatoxins, aflavarins), polyketide-non ribosomal peptides (leporins), indole-diterpenoids (aflatrem) and other metabolites (kojic acid) (Uka et al., 2020). The effects of these metabolites on bees, which have been largely overlooked, may include growth retardation, reduced pupal and adult size, lower fecundity, loss of fertility, mortality, repellency, and genetic changes, as observed in other insects (Wicklow et al., 1994).

Burnside (1930) reported that a toxin contained in the ether extract from liquid cultures of a strain of *A. flavus* could kill adult bees. Later on, the toxicity of aflatoxin B_1_ was evaluated in assays on adult worker bees (Hilldrup and Llewellyn, 1979), revealing a high tolerance towards this compound due to P450-mediated metabolic detoxification (Niu et al., 2011; Johnson et al., 2012). Aflatoxins are major secondary metabolites produced by *Aspergillus* species in the section *Flavi* which are particularly considered for their occurrence as mycotoxins in food products (Cary and Ehrlich, 2006). A wide variation has been observed in the production of aflatoxins and other secondary metabolites in *A. flavus*; at least in part, this plasticity could be influenced by the horizontal transfer of gene clusters encoding biosynthesis of secondary metabolites, a phenomenon which is likely to generally occur in *Aspergillus* species (Pires et al., 2015, Uka et al., 2020).

Aflavarins and aflatrem have been reported for antifeedant and growth reducing effects, respectively, on diverse insect species (TePaske et al., 1992). Leporins form iron complexes which revealed antifeedant and antiinsectan effects on fall armyworms (*Spodoptera frugiperda*), corn earworms (*Helicoverpa zea*) and the Freeman sap beetle (*Carpophilus freemani*) (Cary et al., 2015). Moreover, some *Aspergillus* spp. produce ochratoxins, at varying extents depending on species, strains and growth substrates (Medina et al., 2004), which may exert antifeedant and lethal effect on insects (Paterson et al., 1990). Another secondary metabolite with a putative role as virulence factor is kojic acid, the dominant product in cultures of strains of *A. flavus* (DellaGreca et al., 2019), which may display a regulatory impact on the immune system of honey bees, by interfering with the melanization response, as described in other insects (Shelby and Popham, 2006).

Furthermore, *Aspergillus* species secrete hydrolytic enzymes, which have a role in pathogenesis and digestion of the host tissues. In particular, pectinases and proteinases are important virulence factors involved in plant and insect host colonization, respectively (Leger et al., 2000). The capacity to infect organisms belonging to different kingdoms is widespread among fungi and is influenced by such diverse repertoire of virulence factors. Although *A. flavus* is considered as a saprophytic species, it has been suggested that it can routinely infect both plants and animals with insects acting as vectors (Leger et al., 2000). By infecting pollinator insects, *Aspergillus* spp. can create a very large inoculum to infect flowers and colonize seeds (Klich and Chmielewski, 1985; Leger et al., 2000). However, this hypothesis of a dispersal mechanism exploiting interkingdom host jumps deserves to be examined more in depth.

In general, the role of virulence factors in pathogenesis of *Aspergillus* spp. has yet to be examined, but it is possible that larval mortalities are in part due to toxicity rather than fungal invasion (Foley et al., 2012). Indeed, the stronger virulence displayed by *A. flavus* in honey bees (Foley et al., 2014) matches well with the greater toxicity towards mosquitos of *A. flavus* toxins, as compared to *A. niger* and *A. parasiticus* (Maurya et al., 2011). Such highly toxic and abundant toxins of *A. flavus*, and the fact that *Aspergillus* spp. are opportunistic pathogens with a loose coevolutionary history with bees, may explain the lack of genotypic variation in differently resistant honey bee populations, which has been pointed out in recent studies (Evison et al., 2013, 2016).

## Bee defenses

Opportunistic pathogens, such as *Aspergillus* spp., reveal their pathogenicity only under particular circumstances, especially when host defenses are suppressed or by-passed. Honey bee defenses include physical barriers, immune responses and behavioral responses. Physical barriers against the external invaders are represented by the integument, which covers the bee body, and peritrophic matrix, which protects the midgut (Boucias and Pendland, 2012). Fungal pathogens can adhere to these barriers and penetrate using a mix of physical pressure and lytic enzymes. If these barriers are crossed, honey bees can rely on very efficient cellular and humoral responses (Morfin et al., 2021). Humoral responses include blood clotting and melanization, which are activated, by a proteolytic cascade, when a non-self object is recognized. Fungal cells invading the hemocoel are usually encapsulated and killed by immune cells (hemocytes). Fat body cells synthesize potent antimicrobial peptides which are secreted in the hemolymph, where they act synergistically to kill the remaining microorganisms (Hoffmann, 1995; Ilyasov et al., 2012). This efficient and apparently simple innate immune system is finely regulated through a series of control mechanisms, based on molecular cross-talks and pathways activations (Evans et al., 2006).

However, the main stressors affecting bees, such as the decline in abundance and variety of flowers, the chronic exposure to agrochemicals and the viruses vectored by parasitic mites, negatively impact the immune response (Goulson et al., 2015; Nazzi and Pennacchio, 2018). Deformed wing virus (DWV) is an endemic pathogen which occurs asymptomatically in nearly all hives and can generate an escalating immunosuppression in the infected bees (Brutscher et al., 2015). Considering that immunosuppressed hosts can be more susceptible to pathogens, it should be interesting to study the effect of the interaction between DWV and *Aspergillus* spp. In one of the few studies reporting viral and fungal pathogens co-occurrence in bees, DWV resulted associated with *Aspergillus* in western yellowjacket wasps (*Vespula pensylvanica*) exposed to honey bees infested by *V. destructor* (Loope et al., 2019), suggesting that immune suppression is beneficial to the opportunistic pathogen, as observed in other co-infection studies. Indeed, in mixed infections on ants *Aspergillus* outcompeted *Metarhizium anisopliae*, which is a virulent entomopathogen able to suppress the host’s immune defenses (Hughes and Boomsma, 2004). When defenses are negated, the opportunistic pathogen can supersede the specialized pathogen through a rapid exploitation of host tissues (Boomsma et al., 2014). *Aspergillus*-virus interactions deserve further studies, considering that some honey bee viruses have been recently detected in *A. tubingensis* mycelia and spores, and can be transmitted both horizontally and vertically (Cheng et al., 2022).

Any stress factor competing for metabolic resources may negatively affect immune response and turn an opportunistic pathogen into a deadly invader. The reduction in the availability and diversity of nutritional resources (pollen and nectar) affects immunocompetence (Alaux et al., 2010) and increases susceptibility to *A. flavus*, *A. phoenicis* and *A. fumigatus* (Foley et al., 2012). Honey bee larvae were more susceptible to *A. fumigatus*, when royal jelly is reduced by 20%, highlighting the importance of this component of the larval diet, which contains fundamental nutrients and antimicrobial peptides (Foley et al., 2012; Bílikova et al., 2015).

In response to the selective pressure exerted by the pathogens which affect the hive, honey bees evolved individual and social defenses based on collective actions or on altruistic behaviors performed by infected individuals (Cremer et al., 2007). Bees detect cues of fungal pathogens, avoid direct contact with contaminated individuals, clean the body surface of nestmates by allogrooming, sanitize the nest with antimicrobials and remove dead individuals, reducing the probability of epizootic spread (Cremer et al., 2018). However, behavioral responses, such as the removal of stonebrood infected individuals and the self-isolation of infected bees, which leave the hive by crawling or flying, negatively affect the chance to diagnose the pathology, and may contribute to the underestimation of stonebrood importance (Burnside, 1930; Jensen et al., 2013). Indeed, it is frequently reported that stonebrood has a lower prevalence in the field when compared to chalkbrood, although virulence of *A. flavus* is higher than *A. apis*, with respect to speed of kill and sporulation (Vojvodic et al., 2011; Evison and Jensen, 2018). On the other hand, other *Aspergillus* species, such as *A. tubingensis*, can be considered cryptic pathogens characterized by a low growth rate and can be isolated from chalkbrood mummies (Cheng et al., 2022).

### The role of bee-associated microbiota

Besides intrinsic defense abilities, a relevant role in contrasting fungal infections is reported to derive from symbiotic interactions with other microorganisms (Daisley et al., 2020; Khan et al., 2020). A role in this respect has been inferred for lactic acid bacteria (Janashia et al., 2018; Iorizzo et al., 2021), and for miscellaneous bacteria isolated from honey bee gut (Vojvodic et al., 2011; Borges et al., 2021). In particular, *Apilactobacillus kunkeei*, *Sphingomonas paucimobilis* and *Pseudomonas aeruginosa* showed inhibitory activity against *A. niger,* while cell-free supernatant extracted from culturing strains of *Staphylococcus aureus* and *A. kunkeei* produced inhibitory halo zones around colonies of *A. flavus* (Shehabeldine et al., 2021).

Bee broods supplemented with the acetobacterium *Bombella apis* (formerly known as *Parasaccharibacter apium*) were significantly less infected by *A. flavus* (Miller et al., 2021). Additionally, the presence of this symbiont, known to be associated in the gut and the hypopharyngeal glands, reduced sporulation of *A. flavus* in the few bees that were infected (Miller et al., 2021). Analysis of biosynthetic gene clusters across *B. apis* strains provided indications for their capacity to synthesize antifungal compounds, including a type 1 polyketide, a terpene and an aryl-polyene. The secreted metabolites were effective in suppressing fungal growth, supporting the hypothesis that they mediate fungal inhibition (Miller et al., 2021).

Several methods are under consideration in view of improving the capacity by honey bees to contrast these noxious biotic agents, including the administration of probiotics based on microbial consortia (Borges et al., 2021), or single strains of bacteria and fungi, such as *Aureobasidium melanogenum* (Hsu et al., 2021). Although beneficial fungi may be transient passengers and less important than bacteria as gut symbionts (Decker et al., 2022), they can inhibit growth of other species (Gilliam et al., 1988) and mediate detoxification (Bush et al., 2018), thus enhancing a general honey bee resistance towards pathogens (Yoder et al., 2017).

### Inhibitory effects of bee products

Behaviors that increase sanitation of the nest (Wilson-Rich et al., 2009) include the use of propolis as an antimicrobial against hive pathogens (Bastos et al., 2008). Propolis is a mixture of resinous substances collected from various plants, partially digested by β-glycosidase enzyme of their saliva and added to bee wax to form the final product (Silva et al., 2012). Analysis of propolis of the Australian stingless bee *Tetragonula carbonaria* showed the presence of myrtucommulone and other identified and unidentified alkylated phloroglucinols known for their antibacterial properties (Massaro et al., 2015; Nicoletti et al., 2018). The presence of propolis in all hives acts as a chemical barrier against the establishment of harmful fungi, resulting in the downregulation of immune gene expression, which emphasizes the role of this bee product in disease resistance (Simone et al., 2009).

Propolis and its ethanolic extract (EPE) have been found to inhibit *in vitro* growth and mycotoxin production in *A. flavus* (Ghaly et al., 1998), *A. fumigatus* (Kačániová et al., 2012), *A. parasiticus* (Hashem et al., 2012), and *A. sulphureus* (Pepeljnjak et al., 1982). A more recent study showed that EPE is also able to decrease the expression of genes involved in the aflatoxin biosynthetic pathway (Hosseini et al., 2020). Notably, propolis methanolic extract was shown to promote detoxification of aflatoxin B_1_, as mediated by cytochrome P450 (Niu et al., 2011). Furthermore, dimethylsulfoxide extract of propolis inhibited *A. fumigatus in vitro* (Netíková et al., 2013). In other studies, several organic extracts of propolis proved to be ineffective or have limited efficacy against *A. fumigatus, A. flavus* and *A. niger* (Garedew et al., 2004a; Agüero et al., 2010, 2011, 2014; Kačániová et al., 2012; Falcão et al., 2014). Some extent of inhibition against the same species was also observed to be induced by bee pollen (and beeswax) ethanolic extracts (Kačániová et al., 2012), and by honey against *A. fumigatus, A. flavus, A. parasiticus* and *A. niger* (Wellford et al., 1978; Radwan et al., 1984; Efem and Iwara, 1992; Boukraâ et al., 2008; Tenore et al., 2012; Fahim et al., 2014; Samad et al., 2016). The latter species also proved to be sensitive to honey produced by stingless bees of the genus *Trigona* (Garedew et al., 2004b).

Due to the evident importance of propolis usage, it has been theorized that honey bees may have developed a dependence on the medicinal properties of plant secondary metabolites. Self-medication in honeybees based on the properties of propolis, honey, etc. is still largely unexplored. However, some studies have suggested that honey bee colony declines may depend on the decreased availability of some forage plants with essential medical properties (Tihelka, 2018).

## Environmental fungi, commensals or mutualists

Despite the association with stonebrood, some studies have shown that *Aspergillus* spp., particularly *A. fumigatus, A. flavus* and *A. niger*, equally occur in both diseased and non-diseased colonies (Shoreit and Bagy, 1995). This is not surprising if we consider that the same species includes toxigenic and non-toxigenic strains (Ehrlich, 2014).

*Aspergillus* species are generally considered to be environmentally adaptable, occasionally interacting with their bee hosts. Indeed, they are stress-resistant saprophytes which enter the hive as conidia (resting spores), basically waiting for conditions that favor germination and spoilage of stored pollen (Friedle et al., 2021), which also involves other microbial partners of bee bread (Gilliam et al., 1989; Goerzen, 1991). *Aspergillus* occurrence in honey bee gut may just be the result of pollen ingestion, although further studies are needed to assess if these species can stably colonize bee gut or are just a transient passengers. As matter of fact, spores of *Aspergillus* spp. can germinate at low pH and high temperatures (above 30°C; Araujo and Rodrigues, 2004), which are typical features of honey bee gut/bee bread and hive, respectively.

However, considering that fungal biomass in bee bread increases with storage time (Gilliam et al., 1989; Friedle et al., 2021), we may hypothesize a nutritional benefit resulting in a better fitness for *Aspergillus* species. In this context, *Aspergillus* spp. obtain food without damaging or benefiting bees, which is indicative of a mere association as commensals.

Although a direct evidence of mutualistic symbiosis is lacking, several hints suggest that *Aspergillus* spp. can be beneficial to bees in multiple ways. In fact, they may play a role in competition with pathogenic and/or mycotoxigenic *Aspergillus* species/strains (Bhandari et al., 2020), or produce inhibitory effects towards bee pathogens and parasites (Vojvodic et al., 2011). Moreover, they might enhance honey bee resistance to xenobiotics through detoxification (Berenbaum and Johnson, 2015), or transform and stabilize pollen and bee bread through the production of enzymes, vitamins, antibacterial substances, organic acids and lipids (Kieliszek et al., 2018).

Considering that aflatoxin occurrence in corn and other crops can be deleterious to humans and animals consuming their products, the spread of atoxigenic strains of *A. flavus* has been considered as a possible means to reduce product contamination based on competition with the wild strains. In this context, a field study carried out in Texas showed that using atoxigenic strains of *A. flavus* to replace toxigenic ones has no detrimental effect on the abundance of honey bees and other Apidae belonging to the genera *Ceratina, Diadasia, Melissodes* and *Svastra* (Bhandari et al., 2020). In the field, *A. flavus* is an assemblage of aflatoxigenic and non-aflatoxigenic strains, which lack the ability to produce G-aflatoxins due to a gap in the gene cluster that includes a required cytochrome P450-encoding gene (*cypA*; Ehrlich, 2014). Such equilibrium can be altered by extrinsic factors, such as climate change and fungicide exposure, and intrinsic factors, like genetic recombination derived from sexual reproduction between strains (Ehrlich, 2014). Clearly, these factors have an impact on any biocontrol strategy based on the release of atoxigenic strains (Ehrlich, 2014). Another key issue in such strategies is the absence of a simple method to discriminate between aflatoxigenic and non-aflatoxigenic *Aspergillus* strains (European Food Safety Authority et al., 2022), highlighting the importance of developing sensitive, fast and affordable molecular tools.

The presence of *Aspergillus* spp. in the gut of honey bees seems to be positively correlated with their health status concerning key diseases, such as chalkbrood and American foulbrood, suggesting that lower levels of these fungi may represent a condition of dysbiosis (Gilliam et al., 1988; Ye et al., 2021). Besides representing effective competitors of the chalkbrood fungus *A. apis* (Gilliam et al., 1988; Vojvodic et al., 2011), some *Aspergillus* spp., *A. fumigatus* in particular, can produce antibiotics such as fumagillin, which is used as an effective product against microsporidian pathogens (Bailey, 1953; Guruceaga et al., 2019; Steenwyk et al., 2020; Glavinic et al., 2021). The mutualistic hypothesis is also supported by studies on other insects. As an example, *A. flavus* is helpful to the navel orangeworm (*Amyelois transitella*; Lepidoptera, Pyralidae) in the detoxification of xenobiotics (phytochemicals) added to the artificial diet of larvae reared in the laboratory (Bush et al., 2018).

Considering that *Aspergillus* spp. are mycotoxin producers, toxin extraction, identification and investigation on non-targeted organisms should be performed before their use in biological control. Besides the direct use of fumagillin implying possible effectiveness of natural spread of *A. fumigatus*, so far few studies have directly explored *Aspergillus* spp. as biopesticides against beekeeping pests. *Aspergillus niger* and *A. flavus* have been evaluated as potential biocontrol agents of the small hive beetle (*Aethina tumida*; Coleoptera, Nitidulidae) with limited evidence of efficacy (Sammataro and Yoder, 2011). In the wax moth *G. mellonella*, *A. fumigatus* causes immunosuppression through the production of fumagillin and gliotoxin, which play a critical role in enhancing virulence (Reeves et al., 2004; Fallon et al., 2011).

Fermentation by microorganisms converts stored pollen into bee bread that is fed to honeybee larvae. Although the role of non-aflatoxigenic *Aspergillus* spp. in preserving or enhancing the nutritional value of bee provisions has been poorly investigated, the spread of fungicide use has been suggested as one of the detrimental factors leading to honey bee colony collapse (Yoder et al., 2017). In this context, fungicides negatively affect *Aspergillus* abundance, reducing their beneficial effects. Indeed, bee bread collected from colonies showing chalkbrood symptoms was found to be contaminated by fungicides and contained a reduced number of beneficial fungi, including *Aspergillus* spp. (Yoder et al., 2013). By reducing the abundance of these fungi, fungicides can holistically reduce honey bee immunocompetence and expose the colony to pathogens and parasites.

## Conclusion

**Figure 1:**
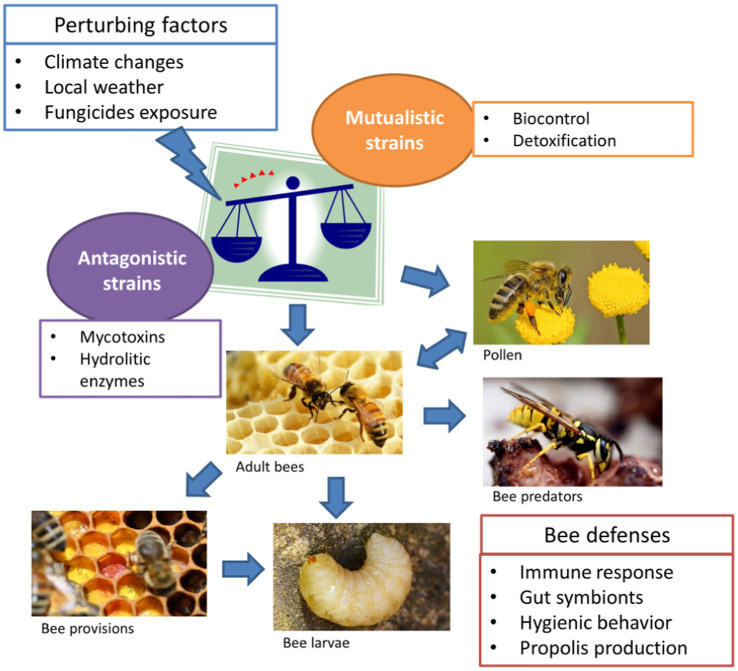
*Aspergillus* spp. infection routes in bees. *Aspergillus* is a species assemblage, including pathogenic strains and atoxigenic strains, which can be perturbed by many extrinsic factors. Such disequilibrium may result in the spread of large inoculums of antagonistic strains, leading to contamination (blue arrows) of pollen, bees and associated species.

Despite the rapid accumulation of documented occurrences of *Aspergillus* spp. in association with wild and domesticated bees in the last decades, the symbiotic relationship between the fungi and pollinators is not clearly defined. The occasional spread of stonebrood counteracts recognized antagonistic properties against some key hive pathogens, which support the conclusion that these mycobiome components are constantly associated to bees at all developmental stages, in a homeostatic equilibrium which is susceptible to be perturbed by several external factors (Figure 1). Current literature supports a dynamic range of symbiotic relationships between *Aspergillus* and bees, from mutualism to antagonism. Antagonistic interactions are basically related to pathogenicity of particular species/strains which are able to produce secondary metabolites acting as virulence factors. Although well documented, stonebrood seems to be underestimated by beekeepers because bees perform hygienic and altruistic behaviors which negatively affect the chance to diagnose the pathology. On the other hand, commensalistic and mutualistic hypotheses have received very little attention. We highlighted diverse beneficial effects of *Aspergillus* presence in the hive: competition with pathogens and parasites, detoxification, stabilization of pollen and bee bread. Regarding the dynamism of such interactions, much of the uncertainty depends on the heterogeneous assemblage of species associated with bees. The recent progresses in techniques for taxonomic identification of fungi have shown that actually this assortment is wider than previously inferred. In fact, common species such as *A. flavus* and *A. niger* have been reconsidered to represent species aggregates including a variable number of taxa, which could perform different ecological roles. Even within a single species, the existence of a variation in the ability to synthesize mycotoxins and other virulence factors might imply different functional relationships with bees, both at individual and colony level.

## Author contributions

RN: conceptualization. AB: data curation. AB and RN: writing—original draft preparation and writing—review and editing. All authors have read and agreed to the published version of the manuscript.

## Funding

This work was supported by received funding from the Ministero dell’Università e della Ricerca, PRIN, project UNICO (2017954WNT, https://www.prin.miur.it). The funders had no role in decision to publish or preparation of the manuscript.

## Conflict of interest

The authors declare that the research was conducted in the absence of any commercial or financial relationships that could be construed as a potential conflict of interest.

## Publisher’s note

All claims expressed in this article are solely those of the authors and do not necessarily represent those of their affiliated organizations, or those of the publisher, the editors and the reviewers. Any product that may be evaluated in this article, or claim that may be made by its manufacturer, is not guaranteed or endorsed by the publisher.

## References

[ref1] Agüero M. B. Gonzalez M. Lima B. Svetaz L. Sanchez M. Zacchino S.. (2010). Argentinean propolis from *Zuccagnia punctata* Cav. (Caesalpinieae) exudates: phytochemical characterization and antifungal activity. J. Agric. Food Chem. 58, 194–201. doi: 10.1021/jf902991t, PMID: 19916546

[ref2] Agüero M. B. Svetaz L. Baroni V. Lima B. Luna L. Zacchino S.. (2014). Urban propolis from San Juan province (Argentina): Ethnopharmacological uses and antifungal activity against Candida and dermatophytes. Ind. Crop. Prod. 57, 166–173. doi: 10.1016/j.indcrop.2014.03.009

[ref3] Agüero M. B. Svetaz L. Sánchez M. Luna L. Lima B. López M. L.. (2011). Argentinean Andean propolis associated with the medicinal plant *Larrea nitida* Cav.(Zygophyllaceae). HPLC–MS and GC–MS characterization and antifungal activity. Food Chem. Toxicol. 49, 1970–1978. doi: 10.1016/j.fct.2011.05.008, PMID: 21600954

[ref4] Alaux C. Ducloz F. Crauser D. Le Conte Y. (2010). Diet effects on honeybee immunocompetence. Biol. Lett. 6, 562–565. doi: 10.1098/rsbl.2009.0986, PMID: 20089536 PMC2936196

[ref5] Alizadeh A. Mossadegh M. S. (1994). Stonebrood and some other fungi associated with *Apis florea* in Iran. J. Apic. Res. 33, 213–218. doi: 10.1080/00218839.1994.11100874

[ref6] Araujo R. Rodrigues A. G. (2004). Variability of germinative potential among pathogenic species of *Aspergillus*. J. Clin. Microbiol. 42, 4335–4337. doi: 10.1128/JCM.42.9.4335-4337.2004, PMID: 15365039 PMC516339

[ref7] Aronstein K. A. Murray K. D. (2010). Chalkbrood disease in honey bees. J. Invertebr. Pathol. 103, S20–S29. doi: 10.1016/j.jip.2009.06.018 19909969

[ref8] Ayo Fasasi K. (2018). Microbiota of honeybees, *Apis mellifera adansonii* (hymenoptera: apidae) from selected ecozones, south west Nigeria. Pak. J. Biol. Sci. 21, 232–238. doi: 10.3923/pjbs.2018.232.238, PMID: 30311486

[ref9] Bailey L. (1953). Effect of fumagillin upon *Nosema apis* (Zander). Nature 171, 212–213. doi: 10.1038/171212a0, PMID: 13036831

[ref10] Bastos E. M. A. F. Simone M. Jorge D. M. Soares A. E. E. Spivak M. (2008). In vitro study of the antimicrobial activity of Brazilian propolis against *Paenibacillus larvae*. J. Invertebr. Pathol. 97, 273–281. doi: 10.1016/j.jip.2007.10.007, PMID: 18054037

[ref11] Batra S. W. T. (1966). Social behavior and nests of some nomiine bees in India (Hymenoptera, halictidæ). Ins. Soc. 13, 145–153. doi: 10.1007/BF02223020

[ref12] Batra L. R. Batra S. W. T. Bohart G. E. (1973). The mycoflora of domesticated and wild bees (Apoidea). Mycopathol. Mycol. Appl. 49, 13–44. doi: 10.1007/BF02057445

[ref13] Batra S. W. T. Bohart G. E. (1969). Alkali bees: response of adults to pathogenic fungi in brood cells. Science 165:607. doi: 10.1126/science.165.3893.607, PMID: 17770861

[ref14] Bayrakal G. Dümen E. Eki̇ci G. Akkaya H. Sezgi̇n F. H. (2020). Detection and molecular examination of pathogens in honey and bees in the northern Marmara region, Turkey. Kafkas. Univ. Vet. Fak. Derg. 26, 313–319. doi: 10.9775/kvfd.2019.22845

[ref15] Bennett J. W. (2010). “An overview of the genus *Aspergillus*,” in Aspergillus: Molecular Biology and Genomics. eds. Machida M. Gomi K. (Wymondham: Caister Academic Press), 1–17.

[ref16] Benoit J. B. Yoder J. A. Sammataro D. Zettler L. W. (2004). Mycoflora and fungal vector capacity of the parasitic mite *Varroa destructor* (Mesostigmata: Varroidae) in honey bee (Hymenoptera: Apidae) colonies. Int. J. Acarol. 30, 103–106. doi: 10.1080/01647950408684376

[ref17] Berenbaum M. R. Johnson R. M. (2015). Xenobiotic detoxification pathways in honey bees. Curr. Opin. Insect Sci. 10, 51–58. doi: 10.1016/j.cois.2015.03.005, PMID: 29588014

[ref18] Bhandari K. B. Longing S. D. West C. P. (2020). Bees occurring in corn production fields treated with atoxigenic *Aspergillus flavus* (Texas, USA). Agronomy 10:571. doi: 10.3390/agronomy10040571

[ref19] Bílikova K. Huang S.-C. Lin I.-P. Šimuth J. Peng C.-C. (2015). Structure and antimicrobial activity relationship of royalisin, an antimicrobial peptide from royal jelly of *Apis mellifera*. Peptides 68, 190–196. doi: 10.1016/j.peptides.2015.03.001, PMID: 25784287

[ref20] Boguś M. I. Wrońska A. K. Kaczmarek A. Boguś-Sobocińska M. (2021). In vitro screening of 65 mycotoxins for insecticidal potential. PLoS One 16:e0248772. doi: 10.1371/journal.pone.0248772, PMID: 33735295 PMC7971479

[ref21] Boomsma J. J. Jensen A. B. Meyling N. V. Eilenberg J. (2014). Evolutionary interaction networks of insect pathogenic fungi. Annu. Rev. Entomol. 59, 467–485. doi: 10.1146/annurev-ento-011613-162054, PMID: 24160418

[ref22] Borges D. Guzman-Novoa E. Goodwin P. H. (2021). Effects of prebiotics and probiotics on honey bees (*Apis mellifera*) infected with the microsporidian parasite *Nosema ceranae*. Microorganisms 9:481. doi: 10.3390/microorganisms9030481, PMID: 33668904 PMC7996622

[ref23] Boucias D. G. Pendland J. C. (2012). Principles of Insect Pathology. New York, NY: Springer Science & Business Media.

[ref24] Boukraâ L. Benbarek H. Ahmed M. (2008). Synergistic action of starch and honey against *Aspergillus niger* in correlation with diastase number. Mycoses 51, 520–522. doi: 10.1111/j.1439-0507.2008.01511.x, PMID: 18331445

[ref25] Bovo S. Utzeri V. J. Ribani A. Cabbri R. Fontanesi L. (2020). Shotgun sequencing of honey DNA can describe honey bee derived environmental signatures and the honey bee hologenome complexity. Sci. Rep. 10:9279. doi: 10.1038/s41598-020-66127-1, PMID: 32518251 PMC7283317

[ref26] Brutscher L. M. Daughenbaugh K. F. Flenniken M. L. (2015). Antiviral defense mechanisms in honey bees. Curr. Opin. Insect. Sci. 10, 71–82. doi: 10.1016/j.cois.2015.04.016 26273564 PMC4530548

[ref27] Bucio Villalobos C. M. López Preciado G. Martínez Jaime O. A. Torres Morales J. J. (2010). Mycoflora associated to bee pollen collected by domesticated bees (*Apis mellifera* L). Nova Sci. 2, 93–103.

[ref28] Burnside C. E. (1930). Fungous Diseases of the Honeybee. United States: Department of Agriculture.

[ref29] Bush D. S. Siegel J. P. Berenbaum M. R. (2018). Accelerated development and toxin tolerance of the navel orangeworm *Amyelois transitella* (Lepidoptera: Pyralidae) in the presence of *Aspergillus flavus*. J. Chem. Ecol. 44, 1170–1177. doi: 10.1007/s10886-018-1027-0, PMID: 30370473

[ref30] Callegari M. Crotti E. Fusi M. Marasco R. Gonella E. De Noni I.. (2021). Compartmentalization of bacterial and fungal microbiomes in the gut of adult honeybees. NPJ Biofilms Microbiomes 7, 42–15. doi: 10.1038/s41522-021-00212-9, PMID: 33963194 PMC8105395

[ref31] Cary J. W. Ehrlich K. C. (2006). Aflatoxigenicity in *Aspergillus*: molecular genetics, phylogenetic relationships and evolutionary implications. Mycopathologia 162, 167–177. doi: 10.1007/s11046-006-0051-8, PMID: 16944284

[ref32] Cary J. W. Uka V. Han Z. Buyst D. Harris-Coward P. Y. Ehrlich K. C.. (2015). An *Aspergillus flavus* secondary metabolic gene cluster containing a hybrid PKS-NRPS is necessary for synthesis of the 2-pyridones, leporins. Fungal Genet. Biol. 81, 88–97. doi: 10.1016/j.fgb.2015.05.010, PMID: 26051490

[ref33] Cheng X. Zhang L. Luo J. Yang S. Deng Y. Li J.. (2022). Two pathogenic fungi isolated from chalkbrood samples and honey bee viruses they carried. Front. Microbiol. 13:843842. doi: 10.3389/fmicb.2022.843842 35495671 PMC9039454

[ref34] Cohen H. McFrederick Q. S. Philpott S. M. (2020). Environment shapes the microbiome of the blue orchard bee, *Osmia lignaria*. Microb. Ecol. 80, 897–907. doi: 10.1007/s00248-020-01549-y, PMID: 32572535

[ref35] Cremer S. Armitage S. A. O. Schmid-Hempel P. (2007). Social immunity. Curr. Biol. 17, R693–R702. doi: 10.1016/j.cub.2007.06.008 17714663

[ref36] Cremer S. Pull C. D. Fürst M. A. (2018). Social immunity: emergence and evolution of colony-level disease protection. Annu. Rev. Entomol. 63, 105–123. doi: 10.1146/annurev-ento-020117-043110, PMID: 28945976

[ref37] Cross E. A. Bohart G. E. (1992). The biology of *Imparipes apicola* (Acari: Scutacaridae) and its relationships to the alkali bee, *Nomia melanderi* (Hymenoptera: Halictidae), and to certain fungi in the bee cell ecosystem. J. Kansas Entomol. Soc. 65, 157–173.

[ref38] Dagenais T. R. T. Keller N. P. (2009). Pathogenesis of *Aspergillus fumigatus* in invasive aspergillosis. Clin. Microbiol. Rev. 22, 447–465. doi: 10.1128/CMR.00055-08, PMID: 19597008 PMC2708386

[ref39] Daisley B. A. Chmiel J. A. Pitek A. P. Thompson G. J. Reid G. (2020). Missing microbes in bees: how systematic depletion of key Symbionts erodes immunity. Trends Microbiol. 28, 1010–1021. doi: 10.1016/j.tim.2020.06.006, PMID: 32680791

[ref40] de Graaf D. C. Brunain M. Jacobs F. J. (2008). Implementation of quality control and biosafety measurements in the diagnosis of honey bee diseases. J. Apic. Res. 47, 151–153. doi: 10.1080/00218839.2008.11101442

[ref41] Decker L. E. San Juan P. A. Warren M. L. Duckworth C. E. Gao C. Fukami T. (2022). Higher variability in fungi compared to bacteria in the foraging honey bee gut. Microb. Ecol. doi: 10.1007/s00248-021-01922-5, PMID: 34997310

[ref42] DellaGreca M. De Tommaso G. Salvatore M. M. Nicoletti R. Becchimanzi A. Iuliano M.. (2019). The issue of misidentification of kojic acid with flufuran in *Aspergillus flavus*. Molecules 24:1709. doi: 10.3390/molecules24091709, PMID: 31052538 PMC6539386

[ref43] Deveza M. V. Keller K. M. Lorenzon M. C. A. Nunes L. M. T. Sales É. O. Barth O. M. (2015). Mycotoxicological and palynological profiles of commercial brands of dried bee pollen. Braz. J. Microbiol. 46:1171. doi: 10.1590/S1517-838246420140316,1176, PMID: 26691478 PMC4704650

[ref44] Disayathanoowat T. Li H. Supapimon N. Suwannarach N. Lumyong S. Chantawannakul P.. (2020). Different dynamics of bacterial and fungal communities in hive-stored bee bread and their possible roles: a case study from two commercial honey bees in China. Microorganisms 8:E264. doi: 10.3390/microorganisms8020264, PMID: 32075309 PMC7074699

[ref45] Dümen E. Akkaya H. Öz G. M. Sezgi̇n F. H. (2013). Microbiological and parasitological quality of honey produced in İstanbul. Turk. J. Vet. Anim. Sci. 37, 602–607. doi: 10.3906/vet-1301-46

[ref46] Efem S. E. E. Iwara C. I. (1992). The antimicrobial spectrum of honey and its clinical significance. Infection 20, 227–229. doi: 10.1007/BF02033065, PMID: 1521889

[ref47] Ehrlich K. (2014). Non-aflatoxigenic *Aspergillus flavus* to prevent aflatoxin contamination in crops: advantages and limitations. Front. Microbiol. 5:50. doi: 10.3389/fmicb.2014.00050 24575088 PMC3918586

[ref48] European Food Safety Authority Alvarez F. Arena M. Auteri D. Binaglia M. Castoldi A. F.. (2022). Peer review of the pesticide risk assessment of the active substance *Aspergillus flavus* strain MUCL54911. EFSA J. 20:e07202. doi: 10.2903/j.efsa.2022.7202 35386571 PMC8968767

[ref49] Evans J. D. Aronstein K. Chen Y. P. Hetru C. Imler J.-L. Jiang H.. (2006). Immune pathways and defence mechanisms in honey bees *Apis mellifera*. Insect Mol. Biol. 15, 645–656. doi: 10.1111/j.1365-2583.2006.00682.x 17069638 PMC1847501

[ref50] Evison S. E. F. Fazio G. Chappell P. Foley K. Jensen A. B. Hughes W. O. H. (2013). Host–parasite genotypic interactions in the honey bee: the dynamics of diversity. Ecol. Evol. 3, 2214–2222. doi: 10.1002/ece3.599 23919163 PMC3728958

[ref51] Evison S. E. F. Fazio G. Chappell P. Foley K. Jensen A. B. Hughes W. O. H. (2016). Innate expression of antimicrobial peptides does not explain genotypic diversity in resistance to fungal brood parasites in the honey bee. Apidologie 47, 206–215. doi: 10.1007/s13592-015-0388-4

[ref52] Evison S. E. Jensen A. B. (2018). The biology and prevalence of fungal diseases in managed and wild bees. Curr. Opin. Insect Sci. 26, 105–113. doi: 10.1016/j.cois.2018.02.010 29764649

[ref53] Fahim H. Dasti J. I. Ali I. Ahmed S. Nadeem M. (2014). Physico-chemical analysis and antimicrobial potential of A pis dorsata, A pis mellifera and Z iziphus jujube honey samples from Pakistan. Asian Pac. J. Trop. Biomed. 4, 633–641. doi: 10.12980/APJTB.4.2014APJTB-2014-0095 25183333 PMC4037657

[ref54] Falcão S. I. Vale N. Cos P. Gomes P. Freire C. Maes L.. (2014). In vitro evaluation of Portuguese propolis and floral sources for antiprotozoal, antibacterial and antifungal activity. Phytother. Res. 28, 437–443. doi: 10.1002/ptr.5013 23722631

[ref55] Fallon J. P. Reeves E. P. Kavanagh K. (2011). (2011). The *Aspergillus fumigatus* toxin fumagillin suppresses the immune response of *Galleria mellonella* larvae by inhibiting the action of haemocytes. Microbiology 157, 1481–1488. doi: 10.1099/mic.0.043786-0 21349977

[ref56] Flores J. M. Gámiz V. Jiménez-Marín Á. Flores-Cortés A. Gil-Lebrero S. Garrido J. J.. (2021). Impact of Varroa destructor and associated pathologies on the colony collapse disorder affecting honey bees. Res. Vet. Sci. 135, 85–95. doi: 10.1016/j.rvsc.2021.01.001 33454582

[ref57] Foley K. Fazio G. Jensen A. B. Hughes W. O. H. (2012). Nutritional limitation and resistance to opportunistic *Aspergillus* parasites in honey bee larvae. J. Invertebr. Pathol. 111, 68–73. doi: 10.1016/j.jip.2012.06.006 22750047

[ref58] Foley K. Fazio G. Jensen A. B. Hughes W. O. H. (2014). The distribution of *Aspergillus* spp. opportunistic parasites in hives and their pathogenicity to honey bees. Vet. Microbiol. 169, 203–210. doi: 10.1016/j.vetmic.2013.11.029 24485932

[ref59] Friedle C. D’Alvise P. Schweikert K. Wallner K. Hasselmann M. (2021). Changes of microorganism composition in fresh and stored bee pollen from Southern Germany. Environ. Sci. Pollut. Res. 28, 47251–47261. doi: 10.1007/s11356-021-13932-4 PMC8692275 33893577

[ref60] Frisvad J. C. Hubka V. Ezekiel C. N. Hong S.-B. Nováková A. Chen A. J.. (2019). Taxonomy of Aspergillus section Flavi and their production of aflatoxins, ochratoxins and other mycotoxins. Stud. Mycol. 93, 1–63. doi: 10.1016/j.simyco.2018.06.001, PMID: 30108412 PMC6080641

[ref61] Gams W. Christensen M. Onions A. H. Pitt J. I. Samson R. A. (1986). “Infrageneric taxa of Aspergillus,” in *Advances in Penicillium and Aspergillus Systematics* NATO ASI Series. eds. Samson R. A. Pitt J. I. (Boston, MA: Springer US), 55–62.

[ref62] Garedew A. Schmolz E. Lamprecht I. (2004a). Microbiological and calorimetric investigations on the antimicrobial actions of different propolis extracts: an in vitro approach. Thermochim. Acta 422, 115–124. doi: 10.1016/j.tca.2004.05.037

[ref63] Garedew A. Schmolz E. Lamprecht I. (2004b). Microcalorimetric investigation on the antimicrobial activity of honey of the stingless bee Trigona spp. and comparison of some parameters with those obtained with standard methods. Thermochim. Acta 415, 99–106. doi: 10.1016/j.tca.2003.06.004

[ref64] Garibaldi L. A. Steffan-Dewenter I. Winfree R. Aizen M. A. Bommarco R. Cunningham S. A.. (2013). Wild pollinators enhance fruit set of crops regardless of honey bee abundance. Science 339, 1608–1611. doi: 10.1126/science.1230200 23449997

[ref65] Geldmann J. González-Varo J. P. (2018). Conserving honey bees does not help wildlife. Science 359, 392–393. doi: 10.1126/science.aar2269 29371456

[ref66] Genersch E. Von D. O. W. Kaatz H. Schroeder A. Otten C. Büchler R.. (2010). The German bee monitoring project: a long term study to understand periodically high winter losses of honey bee colonies. Apidologie 41, 332–352. doi: 10.1051/apido/2010014

[ref67] Ghaly M. F. Ezzat S. M. Sarhan M. M. (1998). Use of propolis and ultragriseofulvin to inhibit aflatoxigenic fungi. Folia Microbiol. 43, 156–160. doi: 10.1007/BF02816502 9721607

[ref68] Gilliam M. Prest D. B. (1972). Fungi isolated from the intestinal contents of foraging worker honey bees, *Apis mellifera*. J. Invertebr. Pathol. 20, 101–103. doi: 10.1016/0022-2011(72)90087-0 4413522

[ref69] Gilliam M. Prest D. B. (1987). Microbiology of feces of the larval honey bee, *Apis mellifera*. J. Invertebr. Pathol. 49, 70–75. doi: 10.1016/0022-2011(87)90127-3

[ref70] Gilliam M. Prest D. B. Lorenz B. J. (1989). Microbiology of pollen and bee bread: taxonomy and enzymology of molds. Apidologie 20, 53–68. doi: 10.1051/apido:19890106

[ref71] Gilliam M. Prest D. B. Morton H. L. (1974). Fungi isolated from honey bees, *Apis mellifera*, fed 2,4-D and antibiotics. J. Invertebr. Pathol. 24, 213–217. doi: 10.1016/0022-2011(74)90013-5 4413522

[ref72] Gilliam M. Taber S. Lorenz B. J. Prest D. B. (1988). Factors affecting development of chalkbrood disease in colonies of honey bees, *Apis mellifera*, fed pollen contaminated with *Ascosphaera apis*. J. Invertebr. Pathol. 52, 314–325. doi: 10.1016/0022-2011(88)90141-3

[ref73] Glare T. R. Harris R. J. Donovan B. J. (1996). *Aspergillus flavus* as a pathogen of wasps, *Vespula* spp., in New Zealand. N. Z. J. Zool. 23, 339–344. doi: 10.1080/03014223.1996.9518093

[ref74] Glavinic U. Stevanovic J. Ristanic M. Rajkovic M. Davitkov D. Lakic N.. (2021). Potential of Fumagillin and *Agaricus blazei* mushroom extract to reduce *Nosema ceranae* in honey bees. Insects 12:282. doi: 10.3390/insects12040282 33806001 PMC8064457

[ref75] Goerzen D. W. (1991). Microflora associated with the alfalfa leafcutting bee, *Megachile rotundata* (Fab) (Hymenoptera: Megachilidae) in Saskatchewan, Canada. Apidologie 22, 553–561. doi: 10.1051/apido:19910508

[ref76] Gois G. C. Carneiro G. G. Rodrigues A. E. Silva E.D. Campos F. S. (2010). Microbiological quality of honey bee *Melipona scutellaris*. PubVet. Vol.4 No.9 pp.unpaginated ref.4. Available at: https://www.cabdirect.org/cabdirect/abstract/20113098147 (Accessed March 1, 2022).

[ref77] González G. Hinojo M. J. Mateo R. Medina A. Jiménez M. (2005). Occurrence of mycotoxin producing fungi in bee pollen. Int. J. Food Microbiol. 105, 1–9. doi: 10.1016/j.ijfoodmicro.2005.05.001 16009441

[ref78] Goulson D. Nicholls E. Botías C. Rotheray E. L. (2015). Bee declines driven by combined stress from parasites, pesticides, and lack of flowers. Science 347:1255957. doi: 10.1126/science.1255957 25721506

[ref79] Grupe A. C. I. Quandt C. A. (2020). A growing pandemic: a review of *Nosema* parasites in globally distributed domesticated and native bees. PLoS Pathog. 16:e1008580. doi: 10.1371/journal.ppat.1008580 32555676 PMC7302437

[ref80] Guruceaga X. Perez-Cuesta U. Abad-Diaz de Cerio A. Gonzalez O. Alonso R. M. Hernando F. L.. (2019). Fumagillin, a mycotoxin of *Aspergillus fumigatus*: biosynthesis, biological activities, detection, and applications. Toxins 12:7. doi: 10.3390/toxins12010007 PMC7020470 31861936

[ref81] Hamzelou J. (2007). Where have all the bees gone? Lancet 370:639. doi: 10.1016/S0140-6736(07)61319-1 17720000

[ref82] Hashem A. Abd-Allah E. F. Alwathnani H. A. (2012). effect of propolis on growth, aflatoxins production and lipid metabolism in *Aspergillus parasiticus* spear. Pak. J. Bot. 44, 1153–1158.

[ref83] Hedtke S. M. Blitzer E. J. Montgomery G. A. Danforth B. N. (2015). Introduction of non-native pollinators can Lead to trans-continental movement of bee-associated fungi. PLoS One 10:e0130560. doi: 10.1371/journal.pone.0130560 26102072 PMC4478036

[ref84] Hilldrup J. L. Llewellyn G. C. (1979). Acute toxicity of the mycotoxin aflatoxin B1 in *Apis Mellifera*. J. Apic. Res. 18, 217–221. doi: 10.1080/00218839.1979.11099972

[ref85] Hoffmann J. A. (1995). Innate immunity of insects. Curr. Opin. Immunol. 7, 4–10. doi: 10.1016/0952-7915(95)80022-0 7772280

[ref86] Hosseini H. M. Hamzeh Pour S. Amani J. Jabbarzadeh S. Hosseinabadi M. Mirhosseini S. A. (2020). The effect of Propolis on inhibition of *Aspergillus parasiticus* growth, aflatoxin production and expression of aflatoxin biosynthesis pathway genes. J Environ Health Sci Engineer 18, 297–302. doi: 10.1007/s40201-020-00467-y PMC7203247 32399241

[ref87] Houbraken J. Kocsubé S. Visagie C. M. Yilmaz N. Wang X.-C. Meijer M.. (2020). Classification of *Aspergillus*, *Penicillium*, *Talaromyces* and related genera (*Eurotiales*): an overview of families, genera, subgenera, sections, series and species. Stud. Mycol. 95, 5–169. doi: 10.1016/j.simyco.2020.05.002 32855739 PMC7426331

[ref88] Hsu C.-K. Wang D.-Y. Wu M.-C. (2021). A potential fungal probiotic *Aureobasidium melanogenum* CK-CsC for the Western honey bee, *Apis mellifera*. J. Fungi 7:508. doi: 10.3390/jof7070508 PMC8306588 34202244

[ref89] Hughes W. O. H. Boomsma J. J. (2004). Let your enemy do the work: within–host interactions between two fungal parasites of leaf–cutting ants. Proc. R. Soc. Lond. Ser. B Biol. Sci. 271, S104–S106. doi: 10.1098/rsbl.2003.0115, PMID: 15101433 PMC1809977

[ref90] Ilyasov R. Gaifullina L. Saltykova E. Poskryakov A. Nikolenko A. (2012). Review of the expression of antimicrobial peptide defensin in honey bees L. J. Apicult. Sci. 56, 115–124. doi: 10.2478/v10289-012-0013-y

[ref91] Inglis G. D. Sigler L. Goette M. S. (1993). Aerobic microorganisms associated with alfalfa leafcutter bees (megachile rotundata). Microb. Ecol. 26, 125–143. doi: 10.1007/BF00177048 24190009

[ref92] Iorizzo M. Testa B. Ganassi S. Lombardi S. J. Ianiro M. Letizia F.. (2021). Probiotic properties and potentiality of *Lactiplantibacillus plantarum* strains for the biological control of Chalkbrood disease. J. Fungi 7:379. doi: 10.3390/jof7050379 PMC8151994 34066127

[ref93] Janashia I. Choiset Y. Jozefiak D. Déniel F. Coton E. Moosavi-Movahedi A. A.. (2018). Beneficial protective role of endogenous lactic acid bacteria against mycotic contamination of honeybee beebread. Probiot. Antimicro. Prot. 10, 638–646. doi: 10.1007/s12602-017-9379-2 29297160

[ref94] Jensen A. B. Aronstein K. Flores J. M. Vojvodic S. Palacio M. A. Spivak M. (2013). Standard methods for fungal brood disease research. J. Apic. Res. 52, 1–20. doi: 10.3896/IBRA.1.52.1.13 PMC3816652 24198438

[ref95] Johnson R. M. Mao W. Pollock H. S. Niu G. Schuler M. A. Berenbaum M. R. (2012). Ecologically appropriate xenobiotics induce cytochrome P450s in *Apis mellifera*. PLoS One 7:e31051. doi: 10.1371/journal.pone.0031051 22319603 PMC3272026

[ref96] Kačániová M. Pavličová S. Haščík P. Kociubinski G. Kńazovická V. Sudzina M.. (2009). Microbial communities in bees, pollen and honey from Slovakia. Acta Microbiol. Immunol. Hung. 56, 285–295. doi: 10.1556/amicr.56.2009.3.7 19789142

[ref97] Kačániová M. Rovná K. Arpášová H. Čuboň J. Hleba L. Pochop J.. (2012). In vitro and in vivo antimicrobial activity of propolis on the microbiota from gastrointestinal tract of chickens. J. Environ. Sci. Health A 47, 1665–1671. doi: 10.1080/10934529.2012.687248 22702827

[ref98] Kaznowski A. Szymas B. Jazdzinska E. Kazimierczak M. Paetz H. Mokracka J. (2005). The effects of probiotic supplementation on the content of intestinal microflora and chemical composition of worker honey bees (*Apis mellifera*). J. Apic. Res. 44, 10–14. doi: 10.1080/00218839.2005.11101139

[ref99] Khan R. A. A. Najeeb S. Hussain S. Xie B. Li Y. (2020). Bioactive secondary metabolites from *Trichoderma* spp. against phytopathogenic fungi. Microorganisms 8:817. doi: 10.3390/microorganisms8060817 PMC7356054 32486107

[ref100] Kieliszek M. Piwowarek K. Kot A. M. Błażejak S. Chlebowska-Śmigiel A. Wolska I. (2018). Pollen and bee bread as new health-oriented products: a review. Trends Food Sci. Technol. 71, 170–180. doi: 10.1016/j.tifs.2017.10.021

[ref101] Klein A.-M. Vaissière B. E. Cane J. H. Steffan-Dewenter I. Cunningham S. A. Kremen C.. (2007). Importance of pollinators in changing landscapes for world crops. Proc. R. Soc. Lond. B Biol. Sci. 274, 303–313. doi: 10.1098/rspb.2006.3721 PMC1702377 17164193

[ref102] Klich M. A. Chmielewski M. A. (1985). Nectaries as entry sites for *Aspergillus flavus* in developing cotton bolls. Appl. Environ. Microbiol. 50, 602–604. doi: 10.1128/aem.50.3.602-604.1985 16346880 PMC238675

[ref103] Kulhanek K. Steinhauer N. Rennich K. Caron D. M. Sagili R. R. Pettis J. S.. (2017). A national survey of managed honey bee 2015–2016 annual colony losses in the USA. J. Apic. Res. 56, 328–340. doi: 10.1080/00218839.2017.1344496

[ref104] Kurtzman C. P. Horn B. W. Hesseltine C. W. (1987). *Aspergillus nomius*, a new aflatoxin-producing species related to *Aspergillus flavus* and *Aspergillus tamarii*. Antonie Van Leeuwenhoek 53, 147–158. doi: 10.1007/BF00393843 3116923

[ref105] Lannutti L. Gonzales F. N. Dus Santos M. J. Florin-Christensen M. Schnittger L. (2022). Molecular detection and differentiation of arthropod, fungal, protozoan, bacterial and viral pathogens of honeybees. Vet. Sci. 9:221. doi: 10.3390/vetsci9050221 35622749 PMC9145064

[ref106] Laursen L. (2015). Wild bees: lone rangers. Nature 521, S62–S63. doi: 10.1038/521S62a 25992676

[ref107] Leger R. J. S. Screen S. E. Shams-Pirzadeh B. (2000). Lack of host specialization in *Aspergillus flavus*. Appl. Environ. Microbiol. 66, 320–324. doi: 10.1128/AEM.66.1.320-324.2000 10618242 PMC91824

[ref108] Leska A. Nowak A. Nowak I. Górczyńska A. (2021). Effects of insecticides and microbiological contaminants on *Apis mellifera* health. Molecules 26:5080. doi: 10.3390/molecules26165080 34443668 PMC8398688

[ref109] Loope K. J. Baty J. W. Lester P. J. Wilson Rankin E. E. (2019). Pathogen shifts in a honeybee predator following the arrival of the Varroa mite. Proc. R. Soc. B Biol. Sci. 286:20182499. doi: 10.1098/rspb.2018.2499 PMC6367166 30963859

[ref110] Macfarlane R. P. (1976). Fungi associated with Bombinae (Apidae: Hymenoptera) in North America. Mycopathologia 59, 41–42. doi: 10.1007/BF00491203

[ref111] Massaro C. F. Smyth T. J. Smyth W. F. Heard T. Leonhardt S. D. Katouli M.. (2015). Phloroglucinols from anti-microbial deposit-resins of Australian stingless bees (*Tetragonula carbonaria*). Phytother. Res. 29, 48–58. doi: 10.1002/ptr.5225 25230727

[ref112] Massi F. P. Penha R. E. S. Cavalcante M. C. Viaro H. P. Silva J. J. D. Ferranti L. D. S.. (2015). Identification of *Aspergillus nomius* in bees visiting Brazil nut flowers. Microbes Environ. 30:273. doi: 10.1264/jsme2.ME14146,275 26063353 PMC4567567

[ref113] Maurya P. Mohan L. Sharma P. Srivastava C. N. (2011). Evaluation of larvicidal potential of certain insect pathogenic fungi extracts against *Anopheles stephensi* and *Culex quinquefasciatus*. Entomological Research 41, 211–215. doi: 10.1111/j.1748-5967.2011.00347.x

[ref114] Medina A. González G. Sáez J. M. Mateo R. Jiménez M. (2004). Bee pollen, a substrate that stimulates ochratoxin a production by *Aspergillus ochraceus* Wilh. Syst. Appl. Microbiol. 27, 261–267. doi: 10.1078/072320204322881880 15046315

[ref115] Miller D. L. Smith E. A. Newton I. L. G. (2021). A bacterial symbiont protects honey bees from fungal disease. mBio 12, e00503–e00521. doi: 10.1128/mBio.00503-21 PMC8262860 34101488

[ref116] Morais P. B. Calaça P. S. S. T. Rosa C. A. (2013). “Microorganisms associated with stingless bees,” in Pot-Honey: A Legacy of Stingless Bees. eds. Vit P. Pedro S. R. M. Roubik D. (New York, NY: Springer), 173–186.

[ref117] Morfin N. Anguiano-Baez R. Guzman-Novoa E. (2021). Honey bee (*Apis mellifera*) immunity. Vet. Clin. N. Am. Food Anim. Pract. 37, 521–533. doi: 10.1016/j.cvfa.2021.06.007 34689918

[ref118] Nazzi F. Pennacchio F. (2018). Honey bee antiviral immune barriers as affected by multiple stress factors: a novel paradigm to interpret colony health decline and collapse. Viruses 10:159. doi: 10.3390/v10040159 PMC5923453 29601473

[ref119] Netíková L. Bogusch P. Heneberg P. (2013). Czech ethanol-free propolis extract displays inhibitory activity against a broad spectrum of bacterial and fungal pathogens. J. Food Sci. 78, M1421–M1429. doi: 10.1111/1750-3841.12230 23915150

[ref120] Nicoletti R. Salvatore M. M. Ferranti P. Andolfi A. (2018). Structures and bioactive properties of myrtucommulones and related acylphloroglucinols from Myrtaceae. Molecules 23:3370. doi: 10.3390/molecules23123370 PMC6321051 30572614

[ref121] Niu G. Johnson R. M. Berenbaum M. R. (2011). Toxicity of mycotoxins to honeybees and its amelioration by propolis. Apidologie 42:79. doi: 10.1051/apido/2010039

[ref122] Norden B. B. Scarbrough A. G. (1982). Predators, parasites, and associates of *Anthophora abrupta* Say (Hymenoptera: Anthophoridae). J. N. Y. Entomol. Soc. 90, 181–185.

[ref123] Paterson R. R. M. Simmonds M. J. S. Kemmelmeier C. Blaney W. M. (1990). Effects of brevianamide a, its photolysis product brevianamide D, and ochratoxin a from two *Penicillium* strains on the insect pests *Spodoptera frugiperda* and *Heliothis virescens*. Mycol. Res. 94, 538–542. doi: 10.1016/S0953-7562(10)80017-6

[ref124] Patiño B. González-Salgado A. González-Jaén M. T. Vázquez C. (2005). PCR detection assays for the ochratoxin-producing *Aspergillus carbonarius* and *Aspergillus ochraceus* species. Int. J. Food Microbiol. 104, 207–214. doi: 10.1016/j.ijfoodmicro.2005.02.011 15967531

[ref125] Pepeljnjak S. Jalsenjak I. Maysinger D. (1982). Growth inhibition of bacillus subtilis and composition of various propolis extracts. Pharmazie 37, 864–865. 6819589

[ref126] Pires R. M. C. Moura S. G. de Filho F. Das C. C. Monte A. M. Pires L. F.. (2015). Evaluation of hygienic-sanitary quality of honey from *Apis mellifera* L. obtained in semi-arid region of Piau, Brazil. AJMR 9, 1806–1813. doi: 10.5897/AJMR2015.7657

[ref127] Quinn O. Gruber M. A. M. Brown R. L. Baty J. W. Bulgarella M. Lester P. J. (2018). A metatranscriptomic analysis of diseased social wasps (*Vespula vulgaris*) for pathogens, with an experimental infection of larvae and nests. PLoS One 13:e0209589. doi: 10.1371/journal.pone.0209589 30596703 PMC6312278

[ref128] Ráduly Z. Szabó L. Madar A. Pócsi I. Csernoch L. (2019). Toxicological and medical aspects of *Aspergillus*-derived mycotoxins entering the feed and food chain. Front. Microbiol. 10:2908. doi: 10.3389/fmicb.2019.02908 31998250 PMC6962185

[ref129] Radwan S. S. El-Essawy A. A. Sarhan M. M. (1984). Experimental evidence for the occurrence in honey of specific substances active against microorganisms. Zentralbl. Mikrobiol. 139, 249–255. doi: 10.1016/S0232-4393(84)80047-5 6475343

[ref130] Reeves E. P. Messina C. G. M. Doyle S. Kavanagh K. (2004). Correlation between gliotoxin production and virulence of *Aspergillus fumigatus* in *Galleria mellonella*. Mycopathologia 158, 73–79. doi: 10.1023/B:MYCO.0000038434.55764.16 15487324

[ref131] Ribani A. Utzeri V. J. Taurisano V. Galuppi R. Fontanesi L. (2021). Analysis of honey environmental DNA indicates that the honey bee (*Apis mellifera* L.) trypanosome parasite *Lotmaria passim* is widespread in the apiaries of the North of Italy. J. Invertebr. Pathol. 184:107628. doi: 10.1016/j.jip.2021.107628 34090931

[ref132] Sainger D. K. Garg A. P. Sharma P. D. (1978). Mycoflora of some pollen grains. Acta Botanica Indica. Vol. 6, 165–168 Available at: https://scholar.google.com/scholar_lookup?title=Mycoflora+of+some+pollen+grains&author=Sainger%2C+D.K.&publication_year=1978 (Accessed March 1, 2022).

[ref133] Salvatore M. M. Nicoletti R. Salvatore F. Naviglio D. Andolfi A. (2018). GC–MS approaches for the screening of metabolites produced by marine-derived *Aspergillus*. Mar. Chem. 206, 19–33. doi: 10.1016/j.marchem.2018.08.003

[ref134] Samad A. Khalid A. Javed K. Sattar S. Rafique N. Rashid M. A.. (2016). Fungicidal activity of honey originating from different phytogeographic regions against *Aspergillus niger and Penicillium chrysogenum*. J. Entomol. Zool. Stud. 4, 339–342.

[ref135] Sammataro D. Yoder J. A. (2011). Honey Bee Colony Health: Challenges and Sustainable Solutions. Boca Raton, FL: CRC Press.

[ref136] Schäfer M. O. Horenk J. Wylezich C. (2022). Molecular detection of *Malpighamoeba mellificae* in honey bees. Vet. Sci. 9:148. doi: 10.3390/vetsci9030148 35324875 PMC8949188

[ref137] Schubert M. Spiegel H. Schillberg S. Nölke G. (2018). Aspergillus-specific antibodies – targets and applications. Biotechnol. Adv. 36, 1167–1184. doi: 10.1016/j.biotechadv.2018.03.016 29608951

[ref138] Schwarz R. S. Huang Q. Evans J. D. (2015). Hologenome theory and the honey bee pathosphere. Curr. Opin. Insect Sci. 10, 1–7. doi: 10.1016/j.cois.2015.04.006 29587997

[ref139] Seyedmousavi S. Guillot J. Arné P. de Hoog G. S. Mouton J. W. Melchers W. J. G.. (2015). *Aspergillus* and aspergilloses in wild and domestic animals: a global health concern with parallels to human disease. Med. Mycol. 53, 765–797. doi: 10.1093/mmy/myv067 26316211

[ref140] Shehabeldine A. M. Hashem A. H. Hasaballah A. I. (2021). Antagonistic effect of gut microbiota of the Egyptian honeybees, *Apis mellifera* L. against the etiological agent of Stonebrood disease. *Int J trop*. Insect Sci. 42, 1357–1366. doi: 10.1007/s42690-021-00654-w

[ref141] Shelby K. S. Popham H. J. R. (2006). Plasma phenoloxidase of the larval tobacco budworm, *Heliothis virescens*, is virucidal. J. Insect Sci. 6:16. doi: 10.1673/2006_06_13.1 PMC2990302 19537988

[ref142] Shoreit M. N. Bagy M. M. K. (1995). Mycoflora associated with stonebrood disease in honeybee colonies in Egypt. Microbiol. Res. 150, 207–211. doi: 10.1016/S0944-5013(11)80058-3

[ref143] Silva J. C. Rodrigues S. Feás X. Estevinho L. M. (2012). Antimicrobial activity, phenolic profile and role in the inflammation of propolis. Food Chem. Toxicol. 50, 1790–1795. doi: 10.1016/j.fct.2012.02.097 22425940

[ref144] Simone M. Evans J. D. Spivak M. (2009). Resin collection and social immunity in honey bees. Evolution 63, 3016–3022. doi: 10.1111/j.1558-5646.2009.00772.x 19619221

[ref145] Sinpoo C. Williams G. R. Chantawannakul P. (2017). Dynamics of fungal communities in corbicular pollen and bee bread. Chiang Mai J. Sci. 44, 1244–1256.

[ref146] Steenwyk J. L. Mead M. E. Knowles S. L. Raja H. A. Roberts C. D. Bader O.. (2020). Variation among biosynthetic gene clusters, secondary metabolite profiles, and cards of virulence across *Aspergillus* species. Genetics 216, 481–497. doi: 10.1534/genetics.120.303549 32817009 PMC7536862

[ref147] Stephen W. P. (1959). Maintaining Alkali Bees Alcalfa Seed Production. *Agricultural Experiment Station, Oregon State college; Corvalis* Bulletin 568:23.

[ref148] Stockmaier S. Stroeymeyt N. Shattuck E. C. Hawley D. M. Meyers L. A. Bolnick D. I. (2021). Infectious diseases and social distancing in nature. Science 371:eabc 8881. doi: 10.1126/science.abc8881 33674468

[ref149] Tenore G. C. Ritieni A. Campiglia P. Novellino E. (2012). Nutraceutical potential of monofloral honeys produced by the Sicilian black honeybees (*Apis mellifera* ssp. sicula). Food Chem. Toxicol. 50, 1955–1961. doi: 10.1016/j.fct.2012.03.067 22497901

[ref150] TePaske M. R. Gloer J. B. Wicklow D. T. Dowd P. F. (1992). Aflavarin and β-Aflatrem: new anti-Insectan metabolites from the sclerotia of *Aspergillus flavus*. J. Nat. Prod. 55, 1080–1086. doi: 10.1021/np50086a008

[ref151] Tihelka E. (2018). The immunological dependence of plant-feeding animals on their host’s medical properties may explain part of honey bee colony losses. Arthropod Plant Interact. 12, 57–64. doi: 10.1007/s11829-017-9553-1

[ref152] Tsang C.-C. Tang J. Y. M. Lau S. K. P. Woo P. C. Y. (2018). Taxonomy and evolution of Aspergillus, Penicillium and Talaromyces in the omics era – past, present and future. Comput. Struct. Biotechnol. J. 16, 197–210. doi: 10.1016/j.csbj.2018.05.003 30002790 PMC6039702

[ref153] Uka V. Cary J. W. Lebar M. D. Puel O. De Saeger S. Diana Di Mavungu J. (2020). Chemical repertoire and biosynthetic machinery of the *Aspergillus flavus* secondary metabolome: a review. Compr. Rev. Food Sci. Food Saf. 19, 2797–2842. doi: 10.1111/1541-4337.12638 33337039

[ref154] Vega F. E. Kaya H. K. (2012). Insect Pathology. New York, N.Y.: Academic Press.

[ref155] Vojvodic S. Jensen A. B. James R. R. Boomsma J. J. Eilenberg J. (2011). Temperature dependent virulence of obligate and facultative fungal pathogens of honeybee brood. Vet. Microbiol. 149, 200–205. doi: 10.1016/j.vetmic.2010.10.001 21050682

[ref156] Wellford T. E. T. Eadie T. Llewellyn G. C. (1978). Evaluating, the inhibitory action of honey on fungal growth, sporulation, and aflatoxin production. Z Lebensm Unters Forch 166, 280–283. doi: 10.1007/BF01127653 685476

[ref157] Wicklow D. T. Dowd P. F. Gloer J. B. (1994). “Antiinsectan effects of Aspergillus metabolites,” in *The Genus Aspergillus: From Taxonomy and Genetics to Industrial Application* Federation of European Microbiological Societies Symposium Series. eds. Powell K. A. Renwick A. Peberdy J. F. (Boston, MA: Springer US), 93–114.

[ref158] Wilson-Rich N. Spivak M. Fefferman N. H. Starks P. T. (2009). Genetic, individual, and group facilitation of disease resistance in insect societies. Annu. Rev. Entomol. 54, 405–423. doi: 10.1146/annurev.ento.53.103106.093301 18793100

[ref159] Ye M.-H. Fan S.-H. Li X.-Y. Tarequl I. M. Yan C.-X. Wei W.-H.. (2021). Microbiota dysbiosis in honeybee (*Apis mellifera* L.) larvae infected with brood diseases and foraging bees exposed to agrochemicals. *Royal Society open*. Science 8:201805. doi: 10.1098/rsos.201805 PMC7890499 33614099

[ref160] Yoder J. A. Jajack A. J. Rosselot A. E. Smith T. J. Yerke M. C. Sammataro D. (2013). Fungicide contamination reduces beneficial fungi in bee bread based on an area-wide field study in honey bee, *Apis mellifera*, colonies. J. Toxic. Environ. Health A 76, 587–600. doi: 10.1080/15287394.2013.798846 23859127

[ref161] Yoder J. A. Nelson B. W. Jajack A. J. Sammataro D. (2017). “Fungi and the effects of fungicides on the honey bee Colony,” in Beekeeping–From Science to Practice. eds. Vreeland R. H. Sammataro D. (Cham: Springer International Publishing), 73–90.

[ref162] Zhang J. Gao D. Li Q. Zhao Y. Li L. Lin H.. (2020). Biodegradation of polyethylene microplastic particles by the fungus *Aspergillus flavus* from the guts of wax moth *Galleria mellonella*. Sci. Total Environ. 704:135931. doi: 10.1016/j.scitotenv.2019.135931 31830656

